# Factors considered in ranking orthopedic shoulder and elbow fellowship applicants: a survey of program directors

**DOI:** 10.1016/j.xrrt.2020.12.001

**Published:** 2021-01-01

**Authors:** Jonathan G. Erickson, Kristina P. Johnson, Brian F. Grogan, Lisa K. Cannada, Paul S. Whiting

**Affiliations:** a Department of Orthopedics and Rehabilitation, University of Wisconsin Hospital and Clinics, Madison, WI, USA; b Hughston Clinic & Novant Health, Jacksonville, FL, USA

**Keywords:** Orthopedics, Program directors, Fellowship, Shoulder and elbow, Interview

## Abstract

**Background:**

Orthopedic fellowship positions continue to be increasingly competitive, with most orthopedic residency graduates pursuing fellowship after completion of residency. Shoulder and elbow fellowship training represents an increasingly competitive and relatively smaller cohort of applicants than other subspecialties; there are only 29 programs with a total of 40 offered positions. The purpose of this survey is to identify and rank factors considered most important by shoulder and elbow fellowship directors when identifying potential fellowship candidates.

**Methods:**

A web-based survey was emailed to all 29 orthopedic shoulder and elbow fellowship directors recognized by American Shoulder and Elbow Surgeons. Demographic information was collected regarding program size, total number of applicants interviewed, and total number of applicants subsequently ranked. The survey also included a list of twelve applicant characteristics which each program director was asked to rank in a sequential order (most important to least). The median score of each factor was calculated, and a weighted score was applied to the top five (of twelve) categories selected by each program. Five points were given to the top-ranked factor. Four points were given to factors ranked 2^nd^, three points to factors ranked 3^rd^, two points to factors ranked 4^th^, and one point to factors ranked 5^th^. The weighted scores were then used to determine the most highly desired applicant characteristics.

**Results:**

Twenty-two of 29 (76%) orthopedic shoulder and elbow fellowship programs responded to the survey. Fourteen of 22 (64%) programs interview 20 or fewer applicants each year. No programs ranked more than 25 applicants. Twelve of 22 (55%) of program directors rated the interview as the most important factor, whereas 6 of 22 (27%) selected letters of recommendation. Based on the weighted score calculation, interviews, letters of recommendation, and personal connections to the applicant/letter writers comprised the top three categories, respectively, and captured 193 of 330 (58%) of the total available points in the weighted score. Strength of shoulder/elbow experience in residency, ties to the geographical area, and comments made regarding technical competence scored among the lowest factors.

**Conclusion:**

Orthopedic shoulder and elbow fellowship directors consistently ranked interviews, letters of recommendation, and personal connection to applicant/letter writer higher than other factors when ranking applicants. This information provides both program directors as well as applicants with important information to consider when navigating the shoulder and elbow fellowship application process.

Orthopedic surgery fellowship positions continue to be increasingly competitive, with most orthopedic residency graduates pursuing fellowship training after completion of residency.^2^^,^^7^ Shoulder and elbow surgery represents a particularly competitive orthopedic subspecialty, in large part due to a limited number of training positions. A 2011 survey of 498 orthopedic residents reported that 91% planned to apply for a fellowship, with only 8% pursuing shoulder and elbow surgery training.^6^ There are 29 programs with a total of 40 training positions offered within the San Francisco Match (SF Match) Shoulder & Elbow fellowship match program. In addition to having a small number of applicants, shoulder and elbow surgery is the most recent subspecialty to be included in the SF match program, with 2017 being the first year. Recent 2018 SF Match statistics show that 47 applicants registered for the shoulder and elbow match, with only a 75% initial match rate (30 of 40 positions).^8^

To accommodate the interest in shoulder and elbow surgery and the number of fellowship applicants, program directors (PDs) often interview a high number of applicants each year. PDs are then faced with the daunting task of considering a multitude of variables when evaluating applicants. The purpose of this survey is to identify those factors considered most important by shoulder and elbow fellowship PDs when identifying fellowship applicants.

## Methods

This study was determined to be exempt from our Health Sciences Institutional Review Board. A web-based survey questionnaire was sent to the PDs of all 29 American Shoulder and Elbow Surgeons recognized shoulder and elbow fellowship programs. PDs were contacted using publicly accessible email addresses found on individual program websites or on the American Shoulder and Elbow Surgeons website. Respondents were asked to indicate their fellowship program size (number of training positions per year) as well as the number of applicants interviewed and ranked each year.

PDs were then asked to consider various factors used to evaluate and ultimately rank shoulder and elbow fellowship applicants. A previously published list of 12 applicant factors was provided, and PDs were asked to rank all 12 factors in order of importance.^1^ The 12 factors were displayed in a random order for each survey recipient. A free-text field was also provided in which PDs could write-in any additional factor(s) they considered important when ranking fellowship applicants. PDs received a total of three emails; one was sent at initial survey release, and reminder emails were sent 2 and 4 weeks after survey release. Results were anonymous, and surveys could only be completed once by each individual recipient. The complete survey is shown in Appendix A.

Completed surveys consisted of a ranked list of all 12 criteria along with any write-in factor(s) and relative rank(s) provided by the survey respondent. Completed surveys were then used to create a weighted score for each of the 12 applicant factors (and any write-in criteria). Using the scale developed by Baweja et al^1^, each applicant factor was assigned a weighted score in accordance with the following scale: 5 points were awarded each time a factor was ranked first, 4 points for each second-place rank, 3 points for each third-place rank, 2 points for each fourth-place rank, and 1 point for each fifth-place rank.

## Results

**Figure 1:**
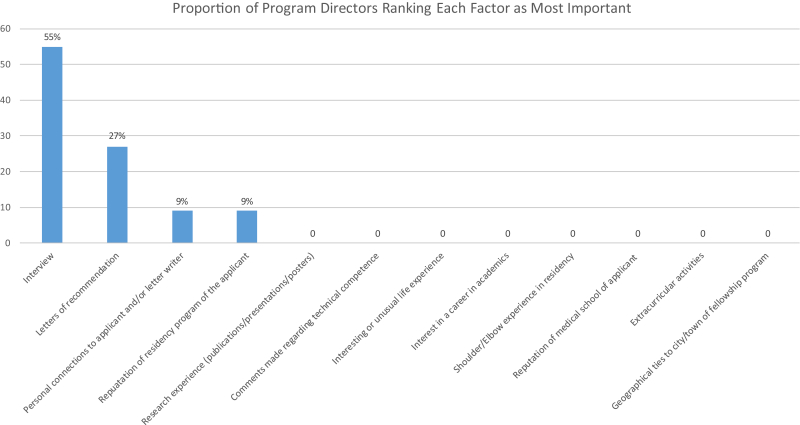
Proportion of responding program directors who ranked each factor as the most important in selecting fellowship applicants.

The overall response rate was 76% (22 of 29 PDs). The vast majority of responding programs 68% (15 of 22) have only a single fellowship position. Fourteen of the 22 responding programs (64%) interview 20 or fewer applicants each year, and no programs reported ranking more than 25 applicants. Of the 22 responding PDs, 12 (55%) rated the interview as the most important factor considered when evaluating fellowship applicants, and 6 (27%) considered letters of recommendation to be the most important factor. The only other factors to receive first place votes included the reputation of the applicant’s residency program and personal connections to the applicant and/or letter writers, which both ranked first by 2 of the 22 responding PDs (9%). Figure 1 shows the top-ranked factors among responding fellowship PDs.

The factors felt to be least important when evaluating shoulder and elbow fellowship applicants included the reputation of the applicant’s medical school (ranked last by 32% of responding PDs) and the applicant’s geographical ties to the fellowship city (ranked last by 18%).

**Figure 2:**
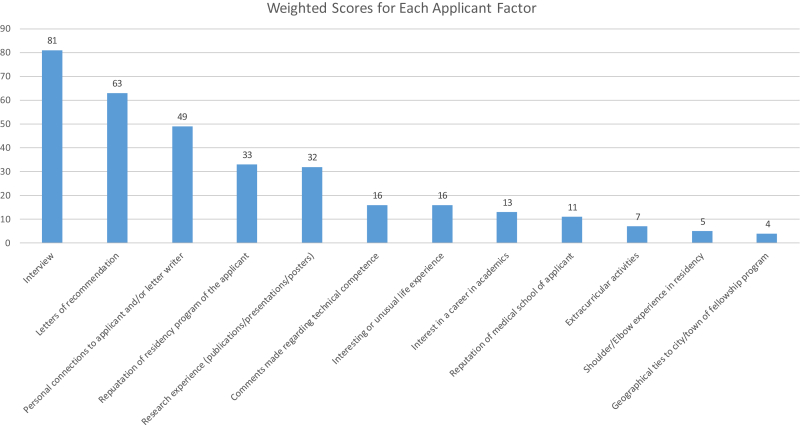
Weighted scores showing the relative importance of various applicant factors in accordance with shoulder and elbow fellowship program directors.

Results of the weighted scores for each applicant factor are shown in Figure 2. Once again, the in-person interview was determined to be the most important applicant factor, with a weighted score of 81. Letters of recommendation (weighted score of 63) and personal connections to applicant/letter writers (weighted score of 49) were considered the second and third most important factors, respectively. These three applicant factors accounted for 193 of the 330 available points (58%) in the weighted score calculation. Strength of the applicant’s shoulder/elbow experience in residency, applicant ties to the geographic area of the fellowship program, and comments made regarding technical competence scored among the lowest applicant factors.

Several PDs identified additional factors they considered important when ranking fellowship applicants. These responses included the following along with their suggested rank by the PD who submitted the response: “Evidence of overcoming hardship/resilience (ranked 1)”, “enthusiasm for the program (ranked 1)” and “ability to communicate well, compassion (to be ranked top 5)”. Other write-in factors, such as the following, were not given a rank but were felt to have an impact when evaluating fellowship applicants: “CV/academic performance, college, med school and residency”, “Interpersonal skills/affective domain”, “honors status, (eg AOA phi beta kappa graduated with honors)” and “Special interest and skills such as business or tech background/ Desire to serve an underserved area.”

## Discussion

Pursuing fellowship training and a career in orthopedic shoulder and elbow surgery is becoming more popular among orthopedic residency graduates.^2^ Given the increasingly competitive and relatively smaller cohort of applicants than other subspecialties, applicants need to be aware of the factors considered most important by PDs when evaluating shoulder and elbow fellowship applicants.

There is limited literature regarding the structure of residency and fellowship interviews within orthopedics.^7^ Programs differ in all aspects of the interview process including the length of the interview day, number of applicants interviewed, number of faculty involved in the interview process, and the elements included in each program’s interview day.^3^^,^^5^ The present study was based on a survey of shoulder and elbow PDs. Although it does not provide program-specific information, it does provide a general overview of the fellowship application process specific to shoulder and elbow. There are 29 shoulder and elbow fellowship programs offering a total of 40 positions. Applicants should be aware that 14 of 22 (64%) of programs rank 15 applicants or fewer. Most programs interview less than 20 applicants, and no programs interview more than 30. In 2018, there were 47 registered applicants within the SF Match system, with an initial match rate of only 75% (30 of 40 positions).^8^ As the match becomes increasingly competitive, this study emphasizes important factors PDs consider before interviewing and ranking shoulder and elbow applicants. The in-person interview was the most important factor considered by PDs. The results suggest that the in-person interaction gives the fellowship directors a better understanding of who the applicants are as individuals and professionals.

Letters of recommendation and personal connections to applicant/letter writer were other highly ranked factors when selecting applicants to interview and rank. These factors are evaluated before the interview. These results underscore the importance of applicants selecting appropriate letter writers and networking with mentors in the field. Applicants with poor shoulder and elbow experience in residency or suboptimal technical competence in shoulder and elbow surgery should not be dissuaded from applying. These factors consistently ranked among the least important.

Strengths of our study include our survey design and methodology, both of which were modeled after studies from other orthopedic subspecialties.^4^^,^^7^ Another notable strength of our study is the high response rate. Nonetheless, our survey may not have identified the full range of factors considered important by PDs. Several PDs utilized the write-in option to indicate other factors they considered important. In addition, responses to the questions in the survey reflect the opinions and preferences of the PD and may not represent those of the other fellowship faculty. However, given the relatively small number of top-ranked factors, we believe our results accurately reflect the most important applicant factors.

In our ranking and weighted scoring system, the interview was clearly the most important factor considered by PDs, but this also highlights another weakness of our study. The interview stands alone on the day that it is conducted, whereas almost all of the other factors can be considered and evaluated before the interview itself. Although unrecognized personal connections, interesting life experience, and geographical ties to a specific area may be harder to ascertain, a general idea can often be elucidated even before the interview. In retrospect, it may have been prudent to take specific aim at factors that affect preinterview selection and compare these factors to postinterview ranking. Despite this potential limitation, however, applicants can still gain valuable information about factors rated highly in the application process.

Shoulder and elbow orthopedic fellowship PDs consider certain factors to be more important than others when ranking fellowship applicants. Our results provide useful information to medical students and residents who plan to pursue fellowship training in shoulder and elbow surgery.

## Conclusion

Orthopedic shoulder and elbow fellowship directors have differing opinions in regard to ranking fellowship applicants, but consistently ranked interviews, letters of recommendation and personal connection to applicant/letter writer higher than factors such as strength of shoulder/elbow experience in residency, ties to geographical area and comments made regarding technical competence. This provides both PDs as well as applicants with additional information to consider when navigating the shoulder and elbow fellowship application process.
