# Supplementary material for: Factors considered in ranking orthopedic shoulder and elbow fellowship applicants: a survey of program directors
Source: JSES Rev Rep Tech. 2021 Jan 1;1(2):151–4. doi: 10.1016/j.xrrt.2020.12.001 (PMC10426661; doi:10.1016/j.xrrt.2020.12.001)
Supplement: Appendix A [file mmc1.pdf]

Appendix A. Complete survey distributed Program Directors

1. How many fellowship positions are available in your program?

1
2
3
4

2. Approximately how many applicants do you interview each year for your fellowship program?

10 or fewer
11-20
21-30
31-40
40 or more

3. Approximately how many fellowship applicants do you rank each year?

10 or fewer
11-15
16-20
21-25
26 or more

4. When ranking the applicants you interviewed, please indicate the most important factors (from the list below) in deciding your rank list. Drag the most important factor to the top of the list and continue doing so until they are listed appropriately from most important (1) to least important (12)

- Geographical ties to the city/town of the fellowship program
- Strength of the applicant's residency training in shoulder and elbow
- Interest in a career in academics
- The interview
- Applicant's research experience (publications/presentations/posters)
- Letters of recommendation
- Personal connections to applicant and/or letter writers
- Extra-curricular activities
- Residency program of applicant
- Medical school of applicant
- Interesting/unusual life experience
- Comments regarding technical competence

5. If there are any additional factors which influence your ranking decision that are not listed, please write in the factors below along with appropriate rank order.
